# Brain Functional Connectivity in the Resting State and the Exercise State in Elite Tai Chi Chuan Athletes: An fNIRS Study

**DOI:** 10.3389/fnhum.2022.913108

**Published:** 2022-06-16

**Authors:** Shilong Wang, Shengnan Lu

**Affiliations:** ^1^Wushu School of China, Beijing Sports University, Beijing, China; ^2^College of Dance and Martial Arts, Capital University of Physical Education and Sport, Beijing, China

**Keywords:** fNIRS, functional correction, resting state, exercise state, elite athletes, tai chi chuan

## Abstract

This study aimed to reveal the characteristics of multi-circuit brain synergy between elite tai chi chuan athletes in resting and exercise states and to provide neuroimaging evidence of improvements in brain function by motor skill training. Functional near-infrared spectroscopy (fNIRS) was used to compare the brain activity of professional tai chi chuan athletes (expert group) and beginners (novice group) in resting and exercise states, and to assess functional connectivity (FC) between the prefrontal lobe and the sensorimotor zone. In the resting state, the FC between the left prefrontal lobe and the right sensorimotor area in the expert group was significantly lower than that in the novice group (*P* < 0.05). In the exercise state, the patterns of FC between the left prefrontal lobe and right sensorimotor area, the right prefrontal lobe and left sensorimotor area, and the left and right sensorimotor areas in the expert group were significantly lower than that in the novice group (*P* < 0.05). From the resting state to the locomotor state, the expert group experienced a greater absolute value of functional connection increment between the left prefrontal cortex and right sensorimotor area, and between the left sensorimotor area and right sensorimotor area (*P* < 0.05). This was positively correlated with the self-evaluation results of motor performance behavior. Under sports conditions, professional athletes’ multi-circuit brain FC strength is significantly reduced, and their elite motor skill performance supports the neural efficiency hypothesis. This may be related to the high adaptation of the brain to specific tasks and the improvement of the integration of somatic perception processing and motor function.

## Introduction

Tai chi chuan is a form of physical and mental exercise that originated in ancient China. It is a combination of a series of rigid and soft, dynamic and static, virtual, physical, and other complex body movements. In the process of tai chi chuan exercise, there are higher requirements for the practitioner’s physical coordination ability and physical and mental integration ability. Therefore, practicing tai chi plays an important role in promoting personal physical health (Hong et al., 2000) and mental health (Gundel et al., 2018). Competitive tai chi is derived from traditional tai chi. It is a competitive style of martial arts formed by adding designated innovative and difficult movements in a Western sport competition system under competition and scoring rules. Excellent tai chi athletes have continuously pursued and emphasized the integrity and standardization of their movements during their long years of training. While their movements are highly artistic, they also have extremely high requirements for physical and mental coordination.

Scientific research into tai chi has been carried out for nearly 40 years, and the direction of exploration has gradually deepened from the skeletal muscle and cardiovascular system to the nervous system. Previous studies on the nervous system mainly focused on middle-aged and elderly people. Long-term tai chi chuan exercises can affect brain structure (Wei et al., 2013; Tao et al., 2017a; Yao et al., 2019) and function (Zou et al., 2008). The improvement of cognitive behavior induced by exercise is mainly manifested in working memory (Diederich et al., 2017; Liu et al., 2019), executive function (Li et al., 2014; Tao et al., 2017b), and the stabilization and regulation of emotions (Liu et al., 2005), and changes in brain plasticity are related to the improvement of cognitive behavior. Unlike the practice common among elderly people, tai chi competitions have extremely high requirements for technical performance and physical stability control. At present, there is little research on the brain function of elite professional tai chi chuan athletes. Studying the neuropsychological mechanism of professional tai chi chuan athletes in the course of special movements is of great significance for understanding the influence of experience and training on brain neural activity, and also helps to reveal the influence of long-term motor skill learning on the cerebral cortex information processing pathway.

In order to study the neural activity of individuals with high-level motor skills, electroencephalography (EEG), functional magnetic resonance imaging (fMRI), or structural magnetic resonance imaging (sMRI) is used to monitor and compare the difference in nerve activity of those with high- and low-level motor skills. There are three types of states analyzed in this study: (1) the resting state (Fong et al., 2014; Wu et al., 2018), which has nothing to do with exercise execution or cognition, and mainly reflects the influence of tai chi exercise on human brain plasticity; (2) immediately after exercise (Liu et al., 2005), which mainly reflects the brain activity during tai chi exercise; and (3) motor cognition (Wayne et al., 2014), which is related to the mental activity of tai chi, and mainly reflects the brain activity during tai chi movement imagination or tacit understanding. Because EEG and MRI are highly sensitive to motion, in order to overcome the interference of motion during signal acquisition, most of the experimental tasks used in this study were in the resting state, and a few EEG studies were performed in the other two task types. However, there may be differences between the neural activity monitored at rest or immediately after exercise and the neural activity during the actual exercise. The exploration of brain activity during exercise is very urgent and necessary, and sports with physical manipulation as the main performance will be useful in such studies.

Functional near-infrared spectroscopy (fNIRS) has become more widely used in various fields. fNIRS is a non-invasive neuroimaging technology that indirectly measures the neural activity of the cerebral cortex through the vascular-nerve coupling mechanism (Tak et al., 2011). Compared with other brain imaging technologies, fNIRS has the advantage of being insensitive to motion artifacts, making it possible to detect brain activity during the actual exercise. In the early stage, fNIRS was used to measure changes in cerebral blood flow in the frontal lobe of two subjects, a veteran practitioner (male, 66 years old) and a beginner (male, 61 years old), during tai chi chuan training. Increased blood flow was observed in the prefrontal region of both subjects (Kokubo et al., 2018). Although the study was preliminary, having included just two subjects, the authors suggest that the phenomenon results from a mental focus on the task declining as subjects become increasingly comfortable with the experiment. Hui et al. recently conducted a comparative study between 23 taijiquan practitioners (age, 65.01 ± 2.61 years) and 32 non-taijiquan practitioners (age, 65.34 ± 2.97 years) to explore the effects of taijiquan on the functional and effector connections of the prefrontal cortex, motor cortex, and occipital cortex. It is believed that long-term participation in tai chi increases the strength of functional connections between the frontal lobe and other brain regions, and changes the interaction between brain regions (Xie et al., 2019). Yang et al. (2020) randomly divided 26 healthy elderly women with no experience in tai chi chuan into the experimental group and the control group. The experimental group underwent tai chi chuan intervention for 8 weeks, while the control group underwent general daily activities. It is believed that taijiquan exercise intervention can significantly improve inhibition control in the elderly.

Previous studies did not focus on the neural activity characteristics of elite tai chi professional athletes, mainly focusing instead on the brain activity of middle-aged and elderly people. In contrast to previous studies, this study is the first to explore the brain FC of elite tai chi professional athletes during exercise. The human brain is a highly complex network. Complex technical actions and mental activities in sports require the coordination of task-related brain regions. Therefore, brain connectivity is used to explore the dynamics of neural signals between different brain regions from the perspective of brain function integration, and then to analyze the synergy of the various brain regions when the human brain performs tasks. Functional connectivity (FC) is defined as the temporal relevance or statistical dependence of neural activities of spatially separated neural units (Friston et al., 1993). When external conditions change, the connections between brain regions will also cause certain changes. It is still unclear whether long-term motor skills and psychological training lead to changes in the coordination of brain regions of tai chi chuan athletes.

Therefore, this study used fNIRS technology to detect brain activity in the resting and tai chi chuan exercise states. The “expert-novice” paradigm was adopted, with national elite tai chi chuan professional athletes as the expert group and non-professional college students as the novice group. This will help us understand the differences in the brain activity of the exercisers with different levels of expertise during specific tasks. After long-term sports skills training, good sports performance is of great significance for revealing the neural mechanism of elite professional athletes.

## Materials and Methods

### Participants

This study recruited 13 elite tai chi chuan athletes as the expert group at Beijing Sport University, and 11 students inexperienced in tai chi chuan as the novice group. There were 13 experts in the expert group (seven males and six females), aged between 20–25 years (average age 22.8 ± 1.64 years), with 10–15 years of tai chi chuan training (average period 11.4 ± 2.76 years). There were 11 subjects in the novice group (six males and five females), aged between 20–25 years (average age 22.3 ± 1.12), with 0.5–1 years of tai chi chuan training (average period 0.7 ± 0.21 years). The details are represented in Table 1. The inclusion criteria were as follows: (1) all subjects could independently practice 24-style simplified tai chi chuan; (2) all subjects were right-handed; (3) no alcohol, staying up late, or heavy exercise 2 days before the experiment; and (4) no history of brain trauma or neurological or mental illness. This study was approved by the sports science experimental ethics committee of Beijing Sport University. All participants provided written informed consent before the start of the study.

**Table 1:** Participant characteristic.

	**Group**		
	**Experiment (*N* = 13)**	**Control (*N* = 11)**	**P**
Age (years)	22.8 ± 1.64	22.3 ± 1.12	0.444
BMI (kg/m)	20.1 ± 0.98	20.7 ± 0.82	0.093
Education (years)	11.2 ± 0.66	10.91 ± 0.67	0.398
Gender (males)	7	6	NA
Tai Chi Chuan duration (years)	11.4 ± 2.76	0.0.7 ± 0.21	0.000
Tai Chi Chuan intensity (hours/week)	10.92 ± 1.21	1.22 ± 0.18	0.000

### Experimental Apparatus

In the experiment, a Nirsmart portable brain function imaging device (Danyang Huichuang Medical Equipment; Danyang, China) was used to monitor blood oxygen signals in local brain regions during resting and exercise tasks. The system collects optical data during human brain activity through near-infrared light with two wavelengths of 760 nm and 850 nm (sampling frequency is 10 Hz). The system consists of 24 emitters and 16 receivers. The detection depth of the photoelectrode was approximately 1.5 cm. Measurement indicators include changes in the concentration of oxygenated hemoglobin (HbO), deoxyhemoglobin (HbR), and total hemoglobin (HbT). HbO has a higher signal-to-noise ratio and a stronger correlation with blood oxygen level dependence (Cui et al., 2010). Therefore, this study mainly uses HbO data for analysis.

### Experimental Design

**Figure 1:**
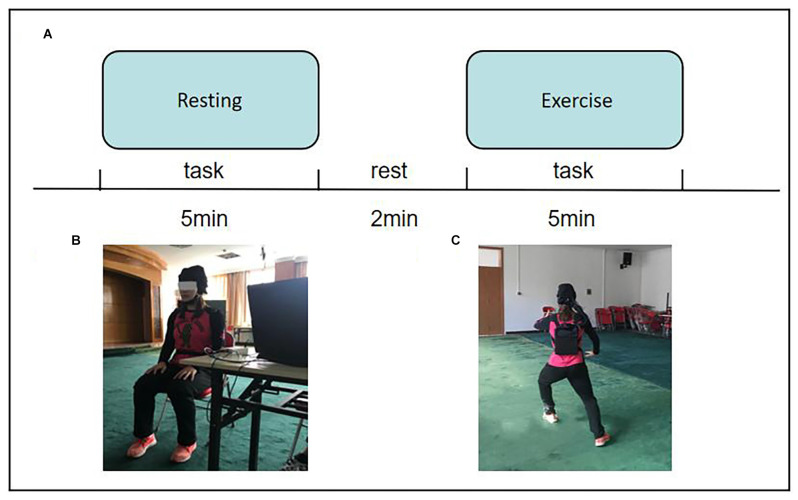
**(A)** Flow chart of experiment tasks. **(B)** Resting state. **(C)** Movement state.

The experimental task settings are shown in Figure 1A. This study adopted a mixed design of 2 (participants; i.e., expert and novice groups) × 2 (task; i.e., resting and exercise state). Resting state (Cai et al., 2018) and exercise state were monitored for a total of 5 min. Ensuring individual monitoring with high ecological validity, a complete set of 24 style tai chi chuan exercises administered over the course of 5 min ensures that individuals are not likely be disturbed while they complete the movements. However, given that tai chi chuan consumes substantial energy, two consecutive sets are likely to reduce motion quality and affect measurements. On the other hand, tai chi chuan and walking represent continuous, dynamic, and complex processes; therefore, fNIRS is particularly well-suited to study them, especially walking (Groff et al., 2019; Suzuki et al., 2004).

The Exercise Performance Self-Assessment Questionnaire was used to evaluate participants’ exercise performance. The questionnaire consists of three factors which respectively measure: (1) task completion; (2) concentration; and (3) kinesthetic perception difficulty. Each factor was structured in the form of four questions, and the questionnaire used a 7-point scale in which participants rated how easy it was to complete the exercise task on a scale of 1 (very difficult) to 7 (very easy). The self-assessment questionnaire was finalized through sampling and testing, and the reliability and validity of the revised self-assessment questionnaire were assessed.

### Experimental Steps

Before the experiment began, participants were familiarized with tasks and procedures. Participants wore portable devices and were asked to sit in a chair and rest for 5 min to eliminate the hemodynamic response caused by other activities before the experiment.

After the start of the formal experiment, the participants first performed the resting state test. The participants were asked to sit still, stay awake, close their eyes, breathe naturally, try their best to maintain a fixed sitting position without moving, and try not to actively perform sexual thinking activities. The test duration was 5 min. After the first task, participants were required to rest for 2 min.

The test scenario is shown in Figures 1B,C.

The second task was the exercise test. The subjects performed a complete set of 24-style tai chi chuan exercises. The exercise duration was controlled to approximately 5 min. If the time was less than 5 min, the second exercise was continued.

Behavior indicators: At the end of the experiment, participants were asked to fill in the Exercise Performance Self-assessment Questionnaire, and the subjects answered the questions they encountered in the process of filling in the questionnaire, returning the evaluation questionnaire in time (the recovery rate was 100%).

### Probe Arrangement

**Figure 2:**
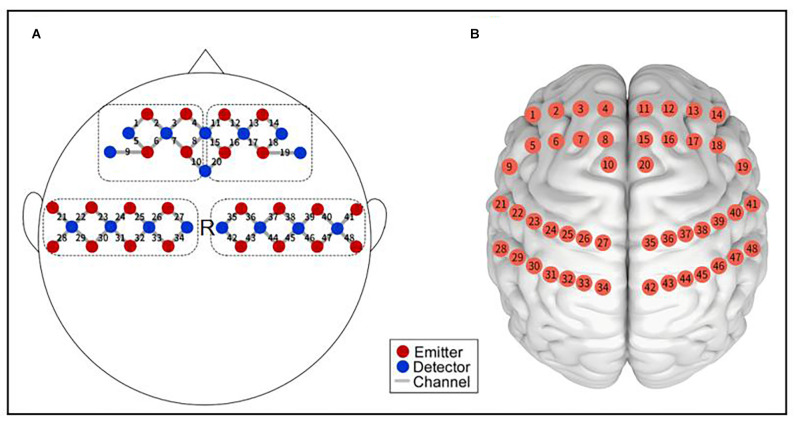
Functional near-infrared spectroscopy measurement. **(A)** Schematic of the imaging pad; 24 emitters (red dots) and 24 detectors (blue dots) were symmetrically placed on the bilateral hemispheres for 48 measurement channels (gray lines), allowing for the measurement of prefrontal and sensorimotor cortices. R was placed in Cz of the international 10–20 system. **(B)** The anatomical position of each measurement channel.

The probe arrangement of the optode cap is shown in Figure 2A. Point R in the figure is the Cz placement point, and optodes were spaced 3 cm apart. The optode cap was composed of three measuring panels. The first panel includes eight emitters and eight receivers, forming 20 channels. The second and third panels each include eight emitters and four receivers, forming 14 channels. A total of 48 measurement channels were used.

Optopolar caps mainly cover the prefrontal cortex (PFC) and sensorimotor cortex (SMC) of the brain regions of interest (ROIs) that this study focused on. The anatomical positions of the measurement channels are shown in Figure 2B. The ROIs corresponding to the channel were divided as follows: 1–10 channels cover the left prefrontal area (LPFC); 11–20 channels cover the right prefrontal area (RPFC); 21–34 channels cover the left sensorimotor area (LSMC); and 35–48 channels cover the right sensorimotor area (RSMC).

### Data Processing

Data analysis was carried out using the MATLAB data processing toolbox (MathWorks, Natick, MA). The following steps were followed to preprocess the experimental data: (1) manually detect the original data and delete channels with oversaturated light intensity; (2) calculate the collected light intensity signal to obtain the change value of the optical density signal; (3) use the spline interpolation method to perform motion artifact correction (Scholkmann et al., 2010), set amplitude and standard deviation thresholds to extract the moving artifact segment, and then model the artifact by a cubic spline interpolation function; (4) use high-pass filtering frequency (set to 0.01 Hz) and low-pass filtering frequency (0.1 Hz) using a bandpass filter to remove system and physiological noise and baseline drift (Zhang et al., 2010; Sasai et al., 2011); and (5) the optical density signal is converted into blood oxygen data reflecting the changes in hemodynamic parameters through the modified Beer-Lambert law (Kocsis et al., 2006). The differential path factor (DPF) is influenced by age and wavelength (Duncan et al., 1995). The DPF of this study was 6.0 based on the length of the adult differential path factor (6.26 ± 14.1%; Scholkmann et al., 2014).

In this study, the Pearson correlation coefficient (R) was used as an indicator to measure the strength of FC (Akin, 2017). The closer the absolute value of R is to 1, the stronger the correlation is. First, the correlation coefficient of HbO concentration between the channels of each subject in the time-series was calculated, and a 48 × 48 FC correlation matrix was obtained. Second, Fisher’s R-to-Z transformation converted the correlation coefficient into Z-values to improve the normality of the data. Then, based on each term, the average value of the FC matrix with a 48 × 48 Z-value was calculated. Finally, the FC matrix with 48 × 48 Z-values was transformed into an FC matrix with 48 × 48 R-values by Fisher’s inverse Z-to-R transformation.

Considering the low spatial resolution of fNIRS, the channel connection strength indices between ROIs were averaged and then statistically analyzed. There were six connection modes among the four ROIs examined in this study: LPFC-RPFC, LPFC-LSMC, LPFC-LSMC, RPFC-RSMC, and LSMC-RSMC.

### Statistical Analysis

Data were analyzed by SPSS Statistics 20.0 (IBM, Somers, USA) statistical software. The descriptive statistics are expressed as “mean ± standard deviation.” The reliability and validity of the questionnaire were tested by cloning a Bach α coefficient, and the internal consistency coefficients of the three subscales were analyzed. Exploratory factor analysis (EFA) was used to test the validity of the questionnaire. The questionnaire results were tested by a two-factor repeated measure ANOVA, the homogeneity of variance was tested by a Mochi sphericity test, and if *P* > 0.05, the intra-subject effect test was used. With *P* < 0.05, a multivariate test was also used. If an interaction was significant, a simple effect analysis was performed.

Neurological results were statistically analyzed and a paired sample t-test was used for the intra-group analysis to compare whether there was a significant difference in functional connection strength between the resting state and operation state (*P* < 0.05). The between groups analysis was carried out using an independent sample t-test, alongside the expert and novice groups in different states (resting state or dynamic) aimed at assessing the change in functional connection strength (*p* < 0.05) across different states (resting state to dynamic; *p* < 0.05). The significance of Pearson correlation coefficient R was corrected by a multiple comparison between ROIs using the FALSE discovery rate (FDR) method (Singh and Dan, 2006). All statistical test results shown in this study were corrected using an FDR.

The normal distribution of the Shapiro–Wilk test data was used to analyze the correlation between behavioral scores and incremental functional connection strength.

## Results

### FC of Resting State

**Figure 3:**
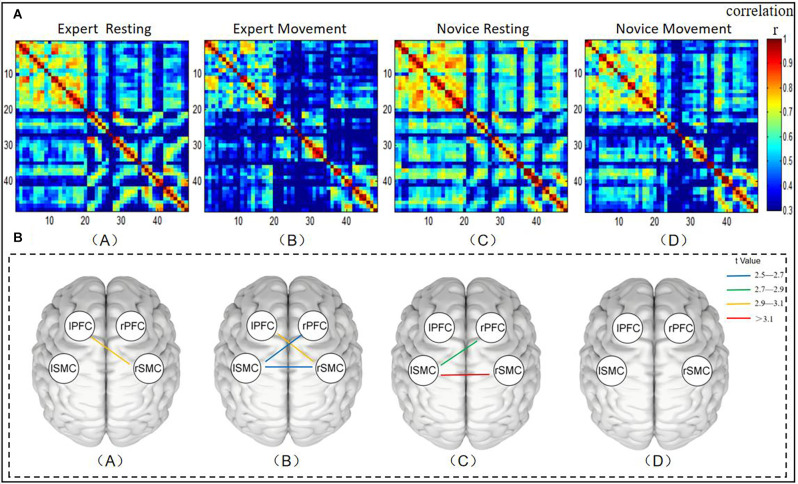
**(A)** Diagram representing the functional connectivity between channels across brain regions. Panel (A) represents the functional connectivity strength of the expert group in its resting state. Panel (B) represents the functional connection strength under the operation of the expert group. Panel (C) indicates the functional connection strength of the novice group in the resting state. Panel (D) indicates the functional connection strength under the dynamic operation of the novice group. The two channels correspond to x- and y-coordinates. The pixel value represents the Pearson correlation coefficient of HbO between the two channels. The gradual change in the color bar from cool to warm colors indicates the change of the Pearson phase relation value, from small to large values. **(B)** Mapping of significant differences in functional connectivity between brain regions. (A) Brain regions with significant differences in functional connectivity between groups at rest. (B) Brain regions with significant differences in functional connectivity across exercise status groups. (C) Brain regions with significant differences between the expert group’s resting state and motility. (D) Brain regions with significant differences between resting state and exercise in the novice group. The color bar color changes from cold to warm colors reflect the t values changing from small to large values.

To observe differences in brain FC between the expert and novice groups in the resting state, Pearson correlation coefficients were used to calculate the correlation between channels, and the FC matrix is shown in Figure 3A (A,C). The FC matrix directly reflects the strength of the FC between channels in the monitored brain regions. The 48 × 48 matrix represents the connection strength between pairs of 48 channels. Because of the symmetry of the FC network, we were only concerned with the FC of the lower trigonometric area of the matrix, and the autocorrelation (diagonal connection) and redundant upper trigonometric parts were not included. It can be seen from the figure that, compared with the novice group, the FC strength between the expert group’s resting state channel decreased.

In order to further evaluate the functional connection between the expert group and the novice group in the resting state, the channel connection strength indicators between the ROIs were averaged before statistical analysis. A comparison of the resting state between groups is shown in Figure 3B (A). As such, brain regions with significant differences in FC in different groups or different states are more intuitively reflected. The line between the two brain regions in the figure shows that there are significant differences in FC between brain regions by comparison (*P* < 0.05). The results show that the correlation coefficients between LPFC-RSMC (*t* = 3.061, *p* = 0.045, df = 22) was significantly different. The FC intensity of the expert group (*r* = 0.387 ± 0.044) in the LPFC-RSMC brain interval was lower than that of the novice group (*r* = 0.483 ± 0.061). Additionally, we also found a decrease in LSMC-RSMC during the resting state. However, no significant changes were observed following thresholding by FDR. In addition, the FC strength of the expert group from the resting state to the moving dynamic channel showed a downward trend.

### FC of Exercise State

The functional connection between the expert group and the novice group under the exercise state was shown in Figure 3A (B,D). It can be seen from the figure that compared with the novice group, the FC strength between the expert group’s exercise state channel decreased. A comparison of transportation performance between groups is shown in Figure 3B (B). The results show that the correlation coefficients among LPFC-RSMC (*t* = 2.929, *p* = 0.0293, df = 22), RPFC-LSMC (*t* = 2.566, *p* = 0.0293, df = 22), and LSMC-RSMC (*t* = 2.578, *p* = 0.0293, df = 22) were significantly different. The dynamic FC intensity of the expert group (*r* = 0.266 ± 0.051) was lower than that of the novice group (*r* = 0.438 ± 0.058) in the LPFC-RSMC brain interval. The dynamic FC intensity of the expert group (*r* = 0.236 ± 0.051) in the RPFC-LSMC brain interval was lower than that of the novice group (*r* = 0.406 ± 0.048). The FC intensity of the expert group (*r* = 0.151 ± 0.041) in the LSMC-RSMC brain interval was lower than that of the novice group (*r* = 0.306 ± 0.038).

### FC Increment From Resting State to Exercise State

First, we compared the performance of resting state and exercise sate within the expert group and the novice group. A comparison between the resting and exercise states in the expert group is shown in Figure 3B (C). The results showed that the correlation coefficients between LPFC-RSMC (*t* = 2.801, *p* = 0.0389, df = 12), LSMC-RSMC (*t* = 3.404, *p* = 0.0187, df = 12) were significantly different. The exercise state FC intensity (*r* = 0.266 ± 0.051) of LPFC-RSMC was lower than that of resting FC intensity (*r* = 0.387 ± 0.044). The FC intensity of LSMC-RSMC during intercerebral movement (*r* = 0.151 ± 0.041) was lower than that at rest (*r* = 0.366 ± 0.053). Figure 3B (D) shows the comparison results of resting state and exercise state in the novice group, and there is no significant difference in connection strength between ROIs after FDR correction.

**Figure 4:**
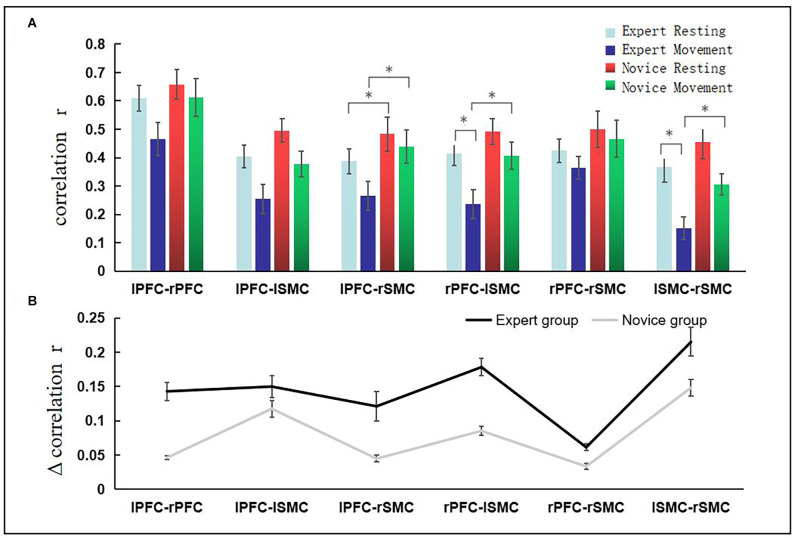
The increment of functional connection intensity of brain interval from resting state to exercise state. Panel **(A)** ^*^(*P* < 0.05) revealed significant differences across ROIs. The abscissa represents the brain area, the ordinate represents the correlation coefficient, the light blue bar represents the resting state of the expert group, the dark blue bar represents the exercise state of the expert group, the red bar represents the resting state of the novice group, and the green color bar indicates the movement state of the novice group. **(B)** The absolute value of functional connectivity (r-value) change is represented from the resting state to the exercise state in each group. Error bars represent standard errors. The black line indicates the expert group, and the gray line indicates the novice group.

As such, we pay more attention to the increment (Δr) of functional connection strength between ROIs when the expert group changes from the resting state to the exercise state, and the absolute value of the increment (Δr) of functional connection strength between ROIs when the novice group changes from the resting state to the operational dynamic state. We calculated the r-value of functional connection strength between ROIs, as shown in Figure 4A, *(*P* < 0.05), at the top of the figure. Figure 4B shows the absolute value of the increment of functional connectivity from resting state to exercise state in the novice group and expert group respectively. The figure indicates that compared with the novice group, the expert group has a larger increment value from the resting state to the exercise state. Statistical tests showed that there were significant differences in the increment of functional connectivity between the expert group and novice group in LPFC-RSMC (*t* = 2.667, *p* = 0.014, df = 22), and LSMC-RSMC (*t* = 3.420, *p* = 0.002, df = 22).

### Correlation Analysis of Behavioral Results and Neural Results

The result of Mockley sphericity test is *P* = 0.923, which satisfies the sphericity hypothesis. The main effect of questionnaire factor was significant *F*_(2,22)_ = 47.791, *p* = 0.000, and the group main effect was significant *F*_(1,22)_ = 159.38, *p* = 0.000. The interaction between groups and questionnaire factors was significant *F*_(2,22)_ = 39.612, *p* = 0.000. The inter-group simple effect results demonstrated that there was no significant difference between the expert group (24.19 ± 1.68) and the novice group (23.51 ± 1.12), *p* = 0.24. The results showed that both the expert group and the novice group could complete the 24-type tai chi chuan exercise task. The concentration score of the expert group (25.71 ± 0.96) was significantly different from that of the novice group (18.41 ± 0.89; *P* = 0.000), and the kinesthetic perceived score of the expert group (22.65 ± 1.92) was significantly different from that of the novice group (17.68 ± 1.01; *P* = 0.000).

**Figure 5:**
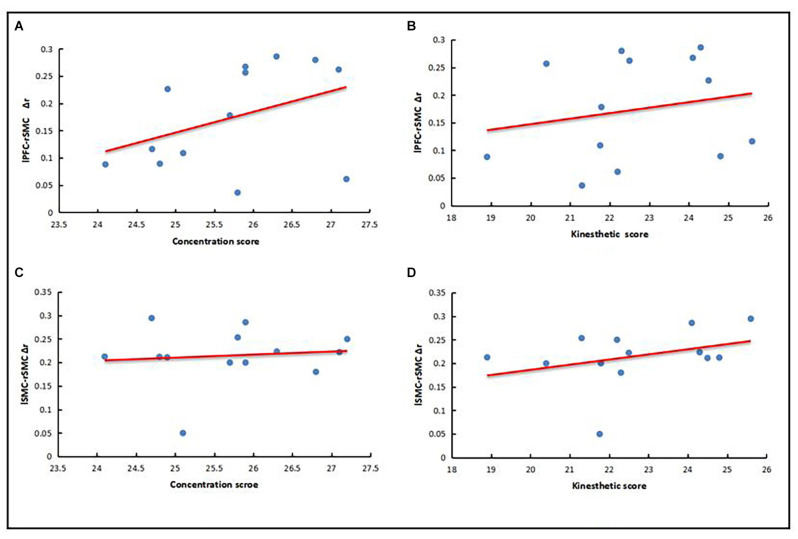
Correlation between behavioral results and neural results. **(A)** LPFC-RSMC (Δr) and concentration score. **(B)** LPFC-RSMC (Δr) and kinesthetic perception score. **(C)** LSMC-RSMC (Δr) and concentration score. **(D)** LSMC-RSMC (Δr) and kinesthetic perception score.

The behavioral results revealed that there were significant differences between the expert group and the novice group in the scores of concentration and kinesthetic perceived, but there were no significant differences in the scores of task completion. Therefore, LPFC-RSMC functional connection strength increment (Δr) and LSMC-RSMC functional connection strength increment (Δr) were taken as the main analysis factors in the expert group, and were correlated with the scores of concentration degree and kinesthetic perception, respectively. Using a Shapiro–Wilk test, the data were found to conform to a normal distribution (*P* > 0.05), and there were no outliers. The results showed that the expert group LPFC-RSMC (Δr) was positively correlated with the concentration score (*r* = 0.4), as represented in Figure 5A. The expert group LPFC-RSMC (Δr) was positively correlated with the score of kinesthetic perception (*r* = 0.21), as is also shown in Figure 5B. The expert group LSMC-RSMC (Δr) was positively correlated with the score of attention concentration (*r* = 0.11), as shown in Figure 5C. The expert group LSMC-RSMC (Δr) was positively correlated with kinesthetic perception score (*r* = 0.35), as reflected in Figure 5D. Therefore, the expert group LPFC-RSMC (Δr) has a high correlation coefficient with the concentration score, and the expert group LSMC-RSMC (Δr) has a high correlation coefficient with the kinesthetic perception score.

## Discussion

### Analysis of the Characteristics of FC in Resting States

The resting state is characterized by the spontaneous activity of brain neurons without a specific cognitive task, which reflects the most essential and inherent characteristics of neural activity. In the resting state, neuronal activity is functionally dependent in the brain region, which can be interpreted as the correlation of the spontaneous fluctuation of blood oxygen signals in the brain region in the time-series. Therefore, the FC of the resting state provides an appropriate window for observing the influence of tai chi chuan on brain function.

The results of the FC comparison between the expert and novice groups in the resting state showed that there were significant differences between LPFC-RSMC (*p* < 0.05). This difference may reflect experience-dependent neuroplasticity, which refers to the ability to change brain structure or function under the action of the external environment or experience (Zilles, 1992). Studies have shown that training is a key factor that induces morphological changes in brain regions and neurons, and changes in network connectivity (Wang et al., 2016). Therefore, we believe that the changes in brain function of the expert group may be related to long-term participation in specific motor skill training.

The results showed that the expert group demonstrated lower FC strength in the frontal and parietal regions compared with the control group. The frontoparietal network (FPN) is believed to be involved in the top-down regulation of the attention system, playing a core role in cognitive control, and participating in the monitoring and resolution of conflicts between anticipation, stimulation, and response. This is similar to the results of previous studies on the brain function of middle-aged and elderly tai chi practitioners (Wei et al., 2017). Compared with those without experience, the low-frequency amplitude of the long-term practitioners in the FPN is reduced and is correlated with cognitive control performance (attention network test). In addition, there is a growing body of literature linking tai chi chuan with the results of mindfulness meditation research. A cross-sectional study (Grant et al., 2011) found that mindfulness meditation specialists showed lower levels of activation in the dorsolateral prefrontal lobe than controls. These studies suggest that the prefrontal and parietal regions play an important role in physical and mental activities (Milton et al., 2007).

Generally speaking, the brain has the ability to realize information integration and separation between brain regions with minimum energy consumption, or the information processing ability with maximum utility. The decrease in FC strength between brain regions reflects either a decrease in the amount of information transmitted or an increase in the efficiency of information processing. Therefore, we speculated that the declining tendency of professional tai chi chuan athletes in the FC of the frontal and parietal lobes may be related to the reduced energy consumption required for cognitive control and the lower attention control load in the resting state. This could be due to the unique movements of tai chi chuan. Tai chi is known as a combination of mental and physical exercises to practice in the process of eliminating distractions and includes dynamic movements, different trajectories of consciousness, or increased focus on certain parts of the body. This state of “one generation mannen thought” can increase awareness and concentration, thus achieving the unity of body and mind in a harmonious state. Over time, it had a positive effect on mood and attention control (Zheng et al., 2015).

### Analysis of the Characteristics of Dynamic FC

The results of this study showed that the dynamic FC between expert and novice groups was significantly different (*p* < 0.05), which mainly manifested as weakened FC strength in the expert group in LPFC-RSMC, RPFC-LSMC, and LSMC-RSMC. Behavioral results revealed that the expert group had higher scores in attention concentration and kinesthetic perception. Moreover, the expert group LPFC-RSMC (Δr) has a high correlation coefficient with the concentration degree score, and the expert group LSMC-RSMC (Δr) has a high correlation coefficient with the kinesthetic perception difficulty score. In this study, because Δr is the absolute value of the change of r-value, the weaker the functional connection strength, the higher the behavior score.

The decrease in FC between brain regions shown by the expert group may be related to the improvement of neural efficiency after long-term intensive training. The “efficiency” hypothesis suggests that with the improvement of skill level, task execution becomes more automated as specific brain regions are devoted to specific neural activities (Debarnot et al., 2015). Thus, behavior is associated with an increase in neural efficiency and corresponds to a reduction in the neural resources involved (Haslinger et al., 2004). Previous studies on the neural characterization of elite basketball players (Qiu et al., 2019) and race-walking athletes (Zhang et al., 2019) have identified the phenomenon of neurological function saving. Professional athletes are more inclined to directly recruit motion-related neuronal activities and can organize the frontal and sensorimotor networks more intensively and effectively, thus inhibiting the irrelevant neural networks, which largely depend on the specific cognitive or motor paradigm of the task. In this study, the athletes had all undergone at least 10 years of professional tai chi training and won titles at the highest national level, and could all be described as elite professional athletes. After a large number of repetitive technical exercises, the expert group mastered 24-style tai chi to a highly standardized and accurate degree of automation. Therefore, we believe that long-term skill training not only improves the movement performance ability of tai chi chuan athletes but also improves the neural work efficiency of the cerebral cortex related to specific tasks. When performing the same motor task, the expert group has more efficient neural network organization and regulates the coordinated operation of limbs. However, the results of this study are inconsistent with those of a study on brain FC in elderly individuals (60–70 years old) during tai chi chuan practice. Compared with tai chi chuan beginners, brain interval FC was enhanced in the Chen tai chi chuan exercise group for more than 5 years (Xie et al., 2019). This may reflect different effects of age or different stages of exercise training on exercise-related brain interval synergy (Halsband and Lange, 2006), and further reasons remain to be explored.

The comparative results within the expert group showed that the functional connections of RPFC-LSMC, and LSMC-RSMC in the brain regions of excellent tai chi chuan athletes were reduced from the resting state to the exercise state. On the one hand, this may be related to the high adaptability of the FPN to multiple task requirements. The FPN has extensive connectivity with the whole brain, and is also known as the “flexible center” (Cole et al., 2013). This suggests that it can achieve multifunctional capabilities by flexibly interacting with a variety of specialized networks in the brain. In this study, The correlation between behavioral results and neural results also demonstrated that the weaker the FC between RPFC-LSMC, the higher the score of concentration during exercise. The decline in FC between brain regions during the transition from a resting state to a motor state may indicate the high adaptability of the FPN to specific tasks, which is explained by the ability of the brain to self-adapt to control its own behavior. This helps us to understand the relationship between superior skill performance and lower brain FC in professional tai chi chuan athletes.

On the other hand, it may be related to the improvement of body perception processing and motor function integration in the sensorimotor area. The SMC region, including the primary motor region of the central anterior gyri (M1), is the center of motion execution and its main function is to transmit motion instructions to effectors. The somatosensory motor region (S1) of the central posterior gyrus plays a key role in processing incoming somatosensory input and helps integrate sensory and motor signals needed for skilled movements and is also important for highly automated movements (Hanakawa et al., 2003). In a study on an elderly population that had been practicing tai chi for a long time, it was also found that compared with the control group, differences in brain structure mainly manifested in the thickening of the central anterior gyrus cortex (Wei et al., 2013). Tai chi chuan is also known as a “perception of movement,” where it is a kind of flexible and progressive dynamic change in the perception of physical activity. A tai chi chuan practitioner is aware of the requirement in the change of the relative position of the body, of muscles contracting or stretching, and joints rotating, and can observe their own feelings more subtly to obtain more accurate proprioception. The correlation between behavioral results and neural results also indicated that the weaker the FC between LSMC-RSMC, the easier the kinesthetic perception during exercise. In addition, the change of the center of gravity in tai chi chuan has higher requirements for coordination and the static and dynamic balance control of the body. Therefore, the advantages of motor skills and the improvement of nerve efficiency in the sensorimotor area exhibited by professional tai chi chuan athletes during exercise may be related to improvements in body perception processing and motor function integration in the sensorimotor area.

## Conclusion

Long-term tai chi training can reduce the synergic effect of the lower frontoparietal brain region, suggesting that professional athletes have lower attention control load in the resting state, which provides new neuroimaging evidence for the plasticity of brain function in tai chi exercise. During exercise, the intensities of intercerebral FC of professional athletes were significantly reduced and the performance of motor skills was elite, supporting the neural efficiency hypothesis.

The improvement of the neural performance of professional athletes while performing tai chi chuan exercises may be related to the brain’s high adaptability to special tasks and the gradual improvement of body perception processing and motor function integration.

## Data Availability Statement

The raw data supporting the conclusions of this article will be made available by the authors, without undue reservation.

## Ethics Statement

The studies involving human participants were reviewed and approved by Sport science experiment ethics committee of Beijing sports university. The patients/participants provided their written informed consent to participate in this study.

## Author Contributions

SW and SL designed the work and wrote the manuscript. SW acquired the data. SL analyzed the data. All authors contributed to the article and approved the submitted version.

## Conflict of Interest

The authors declare that the research was conducted in the absence of any commercial or financial relationships that could be construed as a potential conflict of interest.

## Publisher’s Note

All claims expressed in this article are solely those of the authors and do not necessarily represent those of their affiliated organizations, or those of the publisher, the editors and the reviewers. Any product that may be evaluated in this article, or claim that may be made by its manufacturer, is not guaranteed or endorsed by the publisher.
